# A Multicriteria-Based Comparison of Electric Vehicles Using q-Rung Orthopair Fuzzy Numbers

**DOI:** 10.3390/e25060905

**Published:** 2023-06-06

**Authors:** Sanjib Biswas, Aparajita Sanyal, Darko Božanić, Samarjit Kar, Aleksandar Milić, Adis Puška

**Affiliations:** 1 Decision Science & Operations Management Area, Calcutta Business School, Diamond Harbour Road, Bishnupur Kolkata 743503, West Bengal, India; sanjibb@acm.org; 2 Marketing Area, Calcutta Business School, Diamond Harbour Road, Bishnupur Kolkata 743503, West Bengal, India; aparajitas@calcuttabusinessschool.org; 3 Military Academy, University of Defence in Belgrade, Veljka Lukica Kurjaka 33, 11040 Belgrade, Serbia; milickm5@gmail.com; 4 Department of Mathematics, National Institute of Technology, Durgapur 713209, West Bengal, India; samarjit.kar@maths.nitdgp.ac.in; 5 Department of Public Safety, Government of Brčko District of Bosnia and Herzegovina, Bulevara Mira 1, 76100 Brčko, Bosnia and Herzegovina; adispuska@yahoo.com

**Keywords:** sustainable transportation, electric vehicles, q-rung orthopair fuzzy, entropy method, alternative ranking order method accounting for two-step normalization (AROMAN), Einstein aggregation

## Abstract

The subject of this research is the evaluation of electric cars and the choice of car that best meets the set research criteria. To this end, the criteria weights were determined using the entropy method with two-step normalization and a full consistency check. In addition, the entropy method was extended further with q-rung orthopair fuzzy (qROF) information and Einstein aggregation for carrying out decision making under uncertainty with imprecise information. Sustainable transportation was selected as the area of application. The current work compared a set of 20 leading EVs in India using the proposed decision-making model. The comparison was designed to cover two aspects: technical attributes and user opinions. For the ranking of the EVs, a recently developed multicriteria decision-making (MCDM) model, the alternative ranking order method with two-step normalization (AROMAN), was used. The present work is a novel hybridization of the entropy method, full consistency method (FUCOM), and AROMAN in an uncertain environment. The results show that the electricity consumption criterion (w = 0.0944) received the greatest weight, while the best ranked alternative was A7. The results also show robustness and stability, as revealed through a comparison with the other MCDM models and a sensitivity analysis. The present work is different from the past studies, as it provides a robust hybrid decision-making model that uses both objective and subjective information.

## 1. Introduction

Environmental sustainability is a matter of paramount importance at all levels, such as those of the country, business, and society at large. In order to achieve sustainable development, it is important to consider environmental concerns in economic decisions. Transportation substantially impacts the growth of a country’s gross domestic product (GDP) [1]. However, specific consideration to environmental matters should also be paid. Sustainable transportation is an essential requirement for sustainable development, as transport-based carbon emissions are major causes of air quality problems. A recent report revealed that approximately 16 percent of total greenhouse gases worldwide are contributed by transport vehicles’ toxic fossil fuel emissions [2]. Sustainable transportation is defined as a transportation facility with nondeclining capital, where the capital includes human capital, monetary capital, and natural capital [3,4]. Using electric vehicles (EVs) while enhancing the generation of renewable energies can help reduce the carbon footprint and prevent the depletion of fossil resources [5,6,7,8].

As a consequence, EVs have garnered significant attention from designers and policymakers in the automotive industries, and there has been a substantial increase in the use of EVs over the last few years [9]. EVs are energy efficient and operate with less noise. The Government of India (GOI) has set an ambitious target to migrate to EV production by 2047, with an estimated reduction in oil expenditure of USD 60 billion and a reduction of 37 percent in emissions. Economically, the goal is to curb the over-reliance on crude oil imports to safeguard against currency fluctuations [10,11].

Given the importance of EVs, the extant literature shows an increasing number of contributions in related fields. For instance, Pevec et al. [12] provide a data-driven review of research on EVs from socioeconomic and sociotechnical perspectives to understand the acceptability and usage of EVs and to forecast future trends, estimate the price and capacity requirements, and discuss various issues related to charging station management. The authors observed the need for accurate open data and an appropriate general policy for charging station management. Khazaei [13] aimed to identify the factors influencing Malaysia’s decision to use EVs, mainly battery cars. The author noted the influence of resource requirements, awareness and knowledge, compatibility with existing technologies, and image in society. In this context, researchers [10] have also attempted to examine the influence of the perceived economic benefits on intention to purchase an EV. The authors found that the perceived economic benefits influence the buying decision, positively affecting the mediator variable, such as attitude. Other factors, such as social image and environmental concern, partially affect the decision.

Danielis et al. [14] focused on investigating the mindset of drivers and why they use EVs. The authors observed that price, fuel mileage, and driving range significantly impact drivers’ intentions. The authors also noted the influence of an unexpected variable: free parking. Ziemba [15] compared EVs based on technical, environmental, economic, and social attributes using multicriteria decision-making (MCDM) models and simulations. Singh et al. [16] extended the strand of literature on sustainable transportation and considered carbon emissions, fuel cost, energy efficiency, maintenance, safety, congestion, and noise to compare EVs and found that CO_2_ emissions were the priority criterion. Ziemba [17] adopted a fuzzy and stochastic approach to the EV selection problem from the perspective of consumer expectations. Kumar et al. [18] contemplated past research using a simulation framework and diffusion model to estimate the demand for EVs. The authors elaborated on improvements to charging infrastructure to promote the increased use of EVs. KV et al. [19] also pointed out the dominant effects of financial constraints, performance, charging infrastructure, environmental concerns, and social pressure on the behavioral intentions behind the use EVs. Dixit and Singh [20] put forth a machine learning framework to enfold the predictors of buying decisions and found the influence of demographic variables such as age, income level, and gender, in addition to factors found in past research. On a different note, Srivastava et al. [21] utilized a game theoretic model to demonstrate the need for the government to undertake hybrid tax-subsidy schemes to bolster the use of EVs. Recognizing the influence of charging infrastructure on the adoption of EVs, Koirala, and Tamang [22] found the importance of considering the payback period, land cost, and equipment cost when designing an effective charging system. Hamurcu and Eren [23] applied a lens of MCDM models to compare electric buses. Hezam et al. [24] found a notable impact of the social benefits, infrastructure of charging systems, and incentives of alternative fuel vehicle selection from a sustainability perspective.

In [25], the researchers emphasized the use of energy consumption as a criterion to compare EVs. A heat map and causal association analysis revealed that there was no correlation between energy consumption and charging speed, but there was a significant correlation with range and maximum velocity. In this regard, the authors [26] noted the effect of weather conditions, battery weight, vehicle load, and driving style on variations in the energy consumption.

### 1.1. Subject of Research

From the above discussion, it can be seen that there is growing interest in the identification of user intention to adopt EVs and the technical, managerial, and social factors for successfully embracing this technology and comparing various types of EVs. However, we noticed that the stated field of research is still in the beginning stages, and further exploration is warranted. In addition, we observed that there is an apparent lack of research that concentrates on combining both technical attributes and user intentions to compare EVs. Further, we found that factors such as the user friendliness of the technology and after sales support were not fully considered. These gaps in the literature motivated us to undertake the present work. We aimed to compare a set of 20 leading EVs (namely, electric cars) that are popularly used in emerging markets such as India based on technical attributes and user opinions. As we understand the selection of an EV depends on the satisfactory performance of several attributes or factors, the present work utilized an MCDM framework. Therefore, the current problem is characterized as the selection of the best possible choices through the performance-based ranking of i=1,2, …, m (m=20) alternative options subject to j=1,2, …, n attributes, criteria, or factors. In this study, there were 13 attributes for technical performance and 13 factors for user-opinion-based comparison. The model can be represented as:

$${\left(\begin{array}{ccccc} x_{11} & x_{12} & \ldots & \ldots & x_{1n}\\ x_{21} & x_{22} & \ldots & \ldots & x_{2n}\\ \ldots & \ldots & \ldots & \ldots & \ldots \\ \ldots & \ldots & \ldots & \ldots & \ldots \\ x_{m1} & x_{m2} & \ldots & \ldots & x_{mn} \end{array}\right)}_{m\times n}$$

The present work utilized both objective (secondary data) and subjective information (primary data) for the comparative analysis of the EVs. An objective-information-based model utilizes performance values, which are in the decision matrix, to derive the criteria weights. A subjective-information-based model utilizes user opinions to derive the criteria weights. Both methods have positive and negative aspects. Subjective-opinion-based methods are more flexible in nature, as they take into account the considerations of decision makers, although they are susceptible to subjective bias because of the opinion-based responses. On the other hand, objective-information-based methods do not suffer from this kind of opinion-based bias, but they are limited to the values in the decision matrix [27,28,29]. In this paper, we present a q-rung orthopair fuzzy set (qROFS)-based MCDM framework to offset the effect of imprecise information and uncertainty.

### 1.2. Application of Methods and Research Contribution

A qROFS [30] considers both the degrees of membership (μ) and nonmembership (ϑ), unlike classical fuzzy sets [31]. However, as an added development, unlike intuitionistic fuzzy sets [32] and Pythagorean fuzzy sets [33], it provides decision makers with flexibility in the selection of the values of μ and ϑ by adjusting the value of parameter q so that the limiting condition that the sum of the degree of the membership and nonmembership does not exceed 1 is met (i.e., $\mu^{q}+\vartheta^{q}\leq 1$). Thus, qROFS helps in conducting a more granular analysis with precision while dealing with imprecise information. The extant literature shows increasing applications of qROFS for complex problem solving [34,35,36,37,38,39,40,41,42,43]. In the field of EVs, Deveci et al. [39,41] applied qROFS-based MCDM models to prioritize green transport options and the selection of autonomous vehicles. The preliminary concepts and definitions related to qROFS are provided in Appendix A.

In the field of MCDM, the criteria weights play a very important role. It actually prioritizes the weights of the criteria as per their importance. These criteria weights can be calculated with the two kinds of the information: objective information and subjective information. The present paper used a modified version of the widely used entropy method [44]. The entropy method determines the criteria weights based on the degree of the dispersion of the values. A higher level of dispersion indicates a higher amount of information contained in the corresponding criterion and, thereby, the criterion obtains a higher weight value. The advantage of the entropy method is that it works on asymmetrical information to measure the relative importance of the criteria in terms of their weights. However, despite its wide applications, the entropy method has received criticism. For instance, the presence of too many values of zero in the decision matrix jeopardizes the result. In addition, in many cases, some criteria receive excessively higher weights than the actual degree of differentiation [45]. In this paper, an extension of the classical entropy method is provided using two-step normalization for processing objective information. In addition, the steps of the full consistency method (FUCOM) [46] are infused to obtain a robust computation of the criteria weights. FUCOM provides decision makers with the ability to examine the deviation of the solution from the full consistency value (i.e., DFC) and uses fewer pairwise comparisons (*n* − 1) to better offset the subjective bias. Further, the extended entropy method with q-rung orthopair fuzzy numbers (qROFN) was used for the subjective-opinion-based analysis. Therefore, the proposed full consistent entropy method (F-Entropy) with qROFN provides a robust mechanism to determine the criteria weights, irrespective of the nature of the values in the decision matrix, and is able to offset the subjective bias when dealing with imprecise information. For the ranking of the alternative options, a very recently developed MCDM model, the alternative ranking order method accounting for two-step normalization (AROMAN) [47], was utilized. AROMAN provides the following benefits: use of a linear combination max–min type and vector normalization techniques to provide more flexibility and an accurate representation of the decision matrix through normalization and stable and robust solution. The present paper is a first attempt at technically extending the AROMAN method using qROFN. Further, for the aggregation of user opinions, the ongoing work applies the Einstein aggregation scheme, which further adds novelty to our approach. Researchers (for instance, Ref. [48]) have pointed out several advantages of Einstein aggregation operators such as better approximation than algebraic products and unions.

The advantages of this approach are reflected in more stable decision making, because the application of two normalizations brings stability in the final order of the alternatives. This is because different normalizations lead to different final orders. The use of two normalizations contributes to equalizing the value of the alternative, and based on this, any method can be used without the order of the alternatives remaining stable. This approach is very important for the evaluation of electric cars, because their technical characteristics are similar, which creates a problem for the decision maker. The limitation of this approach is that two normalizations must be calculated, not just one.

The research questions that the current work seeks to answer are:

- What are the factors (technical and user based) that influence the EV selection?
- How can an effective MCDM framework (workable with both objective and subjective information) be developed to address the EV selection problem?
- To what extent doe EVs differ from each other based on technical attributes and user opinions?

The rest of this paper is organized as follows. In Section 2, we describe the methodology, while in Section 3 a summary of the results is presented. Section 4 provides a discussion of the findings while highlighting some of the implications of the research. Finally, Section 5 concludes the work and highlights a future research agenda.

## 2. Materials and Methods

**Figure 1:**
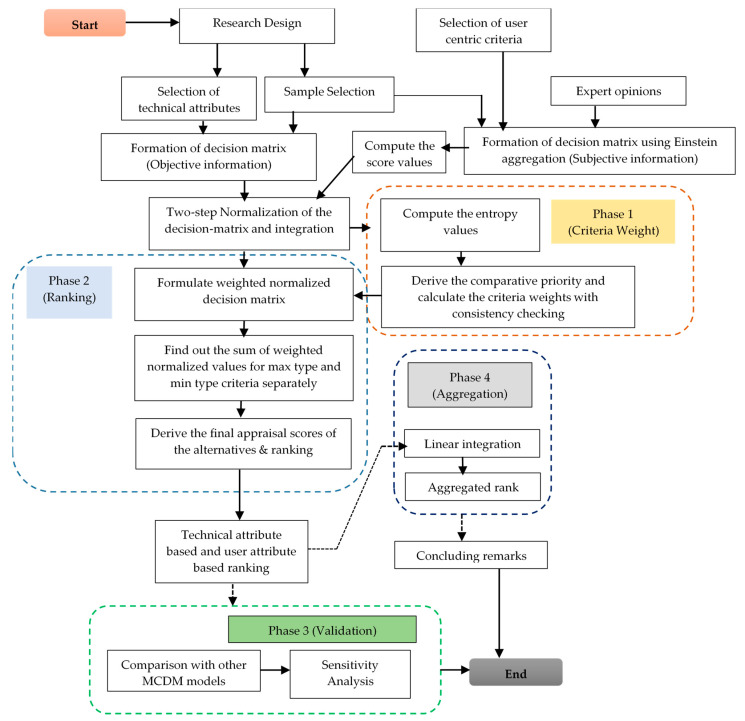
Flowchart of the steps of the research methodology.

The current section provides a step-by-step description of the methodology followed in this paper, which is also pictorially portrayed in Figure 1.

### 2.1. Data

This paper considered the top 20 popular electric cars in India based on the listings available on a commonly used website (https://www.cardekho.com/, accessed on 3 January 2023) [49]. The experts who took part our study mentioned that the website [49] is a popular source for buyers to obtain information related to cars. Let us denote the sample units under comparison as $A_{1},A_{2},A_{3},\;\ldots ,\;A_{20}$. To maintain commercial confidentiality, the actual brand names are not disclosed in this paper. To compare the EVs, we first collected the technical information from the website mentioned above and also from the products’ technical specifications. In the next stage, we collected subjective opinions from three automobile experts regarding users’ ratings on various attributes for comparing the EVs. These experts have substantial experience (more than 15 years) in dealing with the customers and expertise in the technical aspects of the automobile industry including EVs. These three experts possess highly valued auto dealerships that sell EVs manufactured by popular brands over a wide geographical area in the eastern part of India. Hence, it can be justified to consider their opinions as resembling the views of a large number of users.

For the comparison of the EVs on the basis of the technical parameters, the present paper used a set of 13 attributes that were selected in line with the findings of previous research and, subsequently, finalized after expert discussion. The technical attributes are provided in Table 1. Based on the observations made in a previous work, we identified a list of 20 factors that influence buyers’ decisions. Through focus group discussions with the experts, a set of 13 factors from the point of view of customers were finally selected (Table 2), and most of these factors had been considered in previous research. Further to the discussions concerning previous research, we also considered UA2, UA5, and UA9 as influencing factors; these factors are the cornerstones of widely used technology acceptance theory, such as UTAUT theory [50]. The decision matrix for the technical attributes is provided in Appendix B.

For the comparison of the EVs based on the user opinions, the subject experts were requested to rate the EVs with respect to the criteria listed in Table 2. A five-point linguistic qROF scale was used, as shown in Table 3. The ratings of the alternatives by the experts for the criteria used for the comparison are given in Appendix C.

### 2.2. AROMAN Method

The computational steps for the AROMAN method are described below [47].

Step 1. Normalization of the decision matrix.

The AROMAN method uses two schemes for the normalization of the decision matrix: linear max–min and vector normalization. Let $N={\left(n_{ij}\right)}_{m\times n}$ be the normalized decision matrix. The elements $n_{ij}$ can be found as:

(1) $$n_{ij}=\frac{\beta n_{ij(1)}+(1-\beta )n_{ij(2)}}{2};\;i=1,2,\;\ldots ,\;m;\;j=1,2,\;\ldots ,\;n$$

Here, $n_{ij(1)}$ and $n_{ij(2)}$ are the normalized values of the elements of the initial decision matrix, as per Scheme 1 (i.e., linear max–min) and Scheme 2 (i.e., vector normalization), respectively. β is the weighting factor, such that β∈(0,1). As recommended in [47], we took the initial value of β as 0.5. However, β can take any value within the stated range.

(2) $$n_{ij(1)}=\left\{\begin{array}{l} \frac{x_{ij}-x_{j}^{\min}}{x_{j}^{\max}-x_{j}^{\min}};\;j\in j^{+}\\ \frac{x_{j}^{\max}-x_{ij}}{x_{j}^{\max}-x_{j}^{\min}};\;j\in j^{-} \end{array}\right.$$

(3) $$n_{ij(2)}=\frac{x_{ij}}{\sqrt{\sum_{i=1}^{m}x_{ij}^{2}}};\;j=1,2,\;\ldots ,\;n$$

Step 2. Formulation of the weighted normalized decision matrix.

The elements of the weighted normalized decision matrix $V={\left(v_{ij}\right)}_{m\times n}$ can be obtained as:

(4) $$v_{ij}=w_{j}n_{ij};\;i=1,2,\;\ldots ,\;m;\;j=1,2,\;\ldots ,\;n$$

Step 3. Determine the sum of the weighted normalized values for the max-type and min-type criteria, separately.

The sum for the max-type criteria:

(5) $$P_{i}=\sum_{j=1}^{n}v_{ij}{}^{+};\;j\in j^{+};\;i=1,2,\;\ldots ,\;m$$

The sum for the min-type criteria:

(6) $$L_{i}=\sum_{j=1}^{n}v_{ij}{}^{-};\;j\in j^{-};\;i=1,2,\;\ldots ,\;m$$

Step 4. Derive the final appraisal scores of the alternatives:

The final appraisal score for the $i^{th}$ alternative can be obtained using the following definition:

(7) $$S_{i}=L_{i}{}^{\tau }+P_{i}{}^{\left(1-\tau \right)}$$

Here, τ ranges from 0 to 1 and is known as the coefficient degree of the criterion type. As suggested in [47], for the initial case we considered its value to be 0.5.

Decision rule: the higher its value, the better the alternative.

### 2.3. Entropy Method with Full Consistency and Two-Step Normalization

The computational steps are presented below.

Step 1. Standardization of the decision matrix.

Unlike the classical entropy method [44,45], this step involves a two-step normalization, as given by Equations (1)–(3).

Step 2. Calculation of the entropy values of the criteria.

The entropy value for the $j^{th}$ criterion is computed as:

(8) $$E_{j}=-k\sum_{i=1}^{m}n_{ij}\ln(n_{ij})=\frac{-\sum_{i=1}^{m}n_{ij}\ln(n_{ij})}{m}$$

Unlike the classical entropy approach, the weights of the criteria are computed using the following steps taken from the FUCOM model [46] to achieve the full consistency.

Step 3. Ordering of the criteria based on their relative priorities.

We used the entropy values of the criteria to set their relative priorities. The higher the entropy value, the higher the priority.

Let the relative priority order be $C_{j}(1)≻C_{j}(2)≻\ldots ≻C_{j}(r)$, where r denotes the ranking position of a particular criterion. There may be a case where two criteria have the same rank.

Step 4. Establish the comparative priority of the criteria.

The comparative priority (CP) of criteria $C_{j}$ with the $r^{th}$ rank position with respect to the one with the ${\left(r+1\right)}^{th}$ ranking position is denoted as $\phi_{\frac{r}{r+1}}$.

It can be noted that the criterion with *r* = 1 (i.e., at the first position) has the top priority. The other criteria are compared with the criterion with the highest preference. The FUCOM method requires a total of (n−1) pairwise comparisons.

Step 5. Computation of the final weights of the factors.

To calculate the final weights of the criteria, two conditions need to be met:

(9) (a) wrwr+1=ϕrr+1

(10) (b) wrwr+2=ϕrr+1⊗ϕr+1r+2 (mathematical transitivity)

The full consistency is obtained if the deviation from the full consistency (i.e., DFC (χ)) tends to zero. The final model is constructed as:

(11) $$\begin{array}{l} Min\chi \\ s.t\\ \left|\frac{w_{j(r)}}{w_{j(r+1)}}-\phi_{\frac{r}{r+1}}\right|\leq \chi ,\forall j\\ \left|\frac{w_{j(r)}}{w_{j(r+2)}}-\phi_{\frac{r}{r+1}}⊗\phi_{\frac{r+1}{r+2}}\right|\leq \chi ,\forall j\\ \sum w_{j}=1,w_{j}\geq 0,\forall j \end{array}$$

By solving the final model, we obtain the weights for the criteria ($w_{j}$).

### 2.4. qROF-Based Full Consistent Entropy and AROMAN Framework with Einstein Aggregation

Step 1. Aggregation of the opinions of the decision makers given in a qROFN linguistic scale, as provided in Table 3.

Suppose $C_{j}(j=1,2,\;\ldots ,\;n)$ denotes the criteria (where n is finite). In our case, these are the user-centric criteria. $e_{t}(t=1,2,\;\ldots ,\;t)$ is the number of experts. In this case, t=3. $\partial_{qij}^{r}$ is the rating of the $i^{th}$ alternative subject to the $j^{th}$ criterion, given by the $r^{th}$ expert.

Each of the responses received from the experts is a qROFN in nature. Then, by using the qROF Einstein-weighted average (qROFEWA), the aggregated rating (as a qROFN $x_{qij}$) for the $i^{th}$ alternative subject to the $j^{th}$ criterion is obtained as [46]:

(12) $$\begin{array}{l} x_{qij}=qROFEWA\;(\partial^{1}{{}_{q}}_{ij},\partial^{2}{{}_{q}}_{ij},\;\ldots ,\;\partial^{r}{{}_{q}}_{ij})\\ =\langle \begin{array}{l} {\left(\frac{\prod_{t=1}^{r}{\left(1+\mu_{ijt}^{q}\right)}^{\omega_{t}}-\prod_{t=1}^{r}{\left(1-\mu_{ijt}^{q}\right)}^{\omega_{t}}}{\prod_{t=1}^{r}{\left(1+\mu_{ijt}^{q}\right)}^{\omega_{t}}+\prod_{t=1}^{r}{\left(1-\mu_{ijt}^{q}\right)}^{{\overset{-}{\varpi }}_{t}}}\right)}^{\frac{1}{q}}\\ {\left(\frac{2\prod_{t=1}^{r}\vartheta_{ijt}^{\omega_{t}q}}{\prod_{t=1}^{r}{\left(2-\vartheta_{ijt}^{q}\right)}^{\omega_{t}}+\prod_{t=1}^{r}\vartheta_{ijt}^{\omega_{t}q}}\right)}^{\frac{1}{q}} \end{array}\rangle \end{array}$$

Here, $x_{qij}$ is the aggregated rating of the $i^{th}$ alternative subject to the $j^{th}$ criterion (i=1,2, …, m; j=1,2, …, n) and $\omega_{t}$ is the importance of the $t^{th}$ expert. We considered that all of the experts had equal importance. Therefore, after aggregation, we obtained the qROFN-based decision matrix.

Step 2. Obtain the scores of the elements of the qROFN-based decision matrix.

We used the following definition to obtain the score values of the elements of the qROFN-based decision matrix, as given in [54]:

(13) $$x_{ij}=\frac{\left(\mu_{ij}{}^{q}-2\vartheta_{ij}{}^{q}-1\right)}{3}+\frac{\lambda }{3}(\mu_{ij}{}^{q}+\vartheta_{ij}{}^{q}+2);\lambda \in [0,1]$$

Here, λ is a constant scalar value.

Next, the procedural steps of the full consistent entropy method with two-step normalizations were performed to obtain the criteria weights and, thereafter, the computational steps of the AROMAN method were conducted to determine the ranking of the alternatives.

## 3. Results

This section presents step by step the findings of the data analysis. First, the EVs are compared on the basis of their technical performance.

### 3.1. Evaluation of the Performance Based on Technical Attributes

The decision matrix is provided in Appendix B. Table 4 exhibits the elements of the normalized decision matrix, obtained using Equations (1)–(3). As recommended in [47], the initial value for β was taken as 0.5. However, β can take any value within the stated range.

Example of the calculation:

$$\begin{array}{l} n_{11(1)}=\frac{x_{11}-x_{1}^{\min}}{x_{1}^{\max}-x_{1}^{\min}}=\frac{114-90}{1020-90}=0.0258\\ n_{11(2)}=\frac{x_{11}}{\sqrt{\sum_{i=1}^{20}x_{i1}^{2}}}=\frac{114}{\sqrt{5926664}}=0.0468\\ n_{11}=\frac{\beta n_{11(1)}+(1-\beta )n_{11(2)}}{2}=\frac{0.5(0.0258)+(1-0.5)(0.0468)}{2}=0.0182 \end{array}$$

Similarly, the values of all of the other elements of the normalized decision matrix were calculated.

Next, we determined the weights of the criteria using our extended entropy method with two-step normalization and full consistency. Using Equation (8), the entropy values of the criteria were obtained. For example:

$$\begin{array}{l} E_{1}=-k\sum_{i=1}^{20}n_{ij}\ln(n_{ij})=\frac{-\sum_{i=1}^{m}n_{i1}\ln(n_{i1})}{20}=\frac{-(-4.9781)}{20}=1.66174\\ E_{2}=1.58534;\;E_{3}=2.00484;\;E_{4}=1.88651;\;E_{5}=2.10671;\;E_{6}=1.68761;\;E_{7}=2.14716\\ E_{8}=2.10329;\;E_{9}=1.87936;\;E_{10}=2.23583;\;E_{11}=2.31798;\;E_{12}=2.28773;\;E_{13}=2.10065 \end{array}$$

Then, using the entropy values, the comparative priorities of the criteria were set. It can be noted that the higher the entropy value, the higher the information contained in the corresponding criterion. Table 5 provides the weights of the technical attributes using the procedural steps of the FUCOM, as presented in Section 2.3. The final model was derived using Equations (9)–(11). The value of the DFC indicates that there was negligible deviation from the full consistent value despite having 13 criteria. In addition, the criteria weights were rationally distributed (i.e., no apparent outlier). Therefore, the outcome clearly suggests the robustness of the full consistent entropy method.

Final model:

$$\begin{array}{l} Min\chi \\ s.t\\ \left|\frac{w_{11}}{w_{12}}-1.0132\right|\leq \chi ;\left|\frac{w_{12}}{w_{10}}-1.0232\right|\leq \chi ;\left|\frac{w_{10}}{w_{7}}-1.0413\right|\leq \chi ;\left|\frac{w_{7}}{w_{5}}-1.0192\right|\leq \chi ;\left|\frac{w_{5}}{w_{8}}-1.0016\right|\leq \chi ;\\ \left|\frac{w_{8}}{w_{13}}-1.0013\right|\leq \chi ;\left|\frac{w_{13}}{w_{3}}-1.0478\right|\leq \chi ;\left|\frac{w_{3}}{w_{4}}-1.0627\right|\leq \chi ;\left|\frac{w_{4}}{w_{9}}-1.0038\right|\leq \chi ;\left|\frac{w_{9}}{w_{6}}-1.1136\right|\leq \chi ;\\ \left|\frac{w_{6}}{w_{1}}-1.0156\right|\leq \chi ;\left|\frac{w_{1}}{w_{2}}-1.0482\right|\leq \chi ;\\ \left|\frac{w_{11}}{w_{10}}-1.0367\right|\leq \chi ;\left|\frac{w_{12}}{w_{7}}-1.0655\right|\leq \chi ;\left|\frac{w_{10}}{w_{5}}-1.0613\right|\leq \chi ;\left|\frac{w_{7}}{w_{8}}-1.0209\right|\leq \chi ;\left|\frac{w_{5}}{w_{13}}-1.0029\right|\leq \chi ;\\ \left|\frac{w_{8}}{w_{3}}-1.0491\right|\leq \chi ;\left|\frac{w_{13}}{w_{4}}-1.1135\right|\leq \chi ;\left|\frac{w_{3}}{w_{9}}-1.0668\right|\leq \chi ;\left|\frac{w_{4}}{w_{6}}-1.1179\right|\leq \chi ;\left|\frac{w_{9}}{w_{1}}-1.1310\right|\leq \chi ;\\ \left|\frac{w_{6}}{w_{2}}-1.0645\right|\leq \chi ; \end{array}$$

Using the weights of the technical attributes, the final ranking of the EVs was determined. The computational steps of the AROMAN method, as given by Equations (4)–(7), were applied. Table 6 exhibits the weighted normalized decision matrix, and Table 7 shows the final appraisal scores and ranking of the EVs.

For example:

$$\begin{array}{l} v_{11}=w_{1}n_{11}=0.06390\times 0.0182=0.0012\\ P_{1}=\sum_{j=1}^{n}v_{1j}{}^{+};\;j\in j^{+}\\ =v_{11}+v_{12}+v_{13}+\ldots +v_{112}\\ =0.0012+0.0018+\ldots +0.0272=0.1371 \end{array}$$

It can be noted that TA_1_ to TA_12_ are the criteria of the max type. Here, only TA_13_ is a criterion of the min type. Therefore:

$$\begin{array}{l} L_{1}=\sum_{j=1}^{n}v_{1j}{}^{-};\;j\in j^{-}=v_{113}=0.0201\\ S_{1}=L_{1}{}^{\tau }+P_{1}{}^{\left(1-\tau \right)}={\left(0.0201\right)}^{0.5}+{\left(0.1371\right)}^{\left(1-0.5\right)}=0.5119 \end{array}$$

Here, τ ranges from 0 to 1 and is known as a coefficient degree of the criterion type. As suggested in [47] for the initial case, its value is considered as 0.5.

### 3.2. Evaluation of the Performance Based on Users’ Opinions

In stage 2, the EVs were compared based on the opinions of users in a group decision-making environment utilizing an integrated framework of qROF-entropy with two-step normalization and AROMAN with Einstein aggregation. In the following, the findings at various steps of the process are presented.

First, we collected the opinions of the three experts, as provided in Appendix C. Next, the Einstein aggregation (EA) operation was applied to obtain an aggregated rating of each EV with respect to each criterion. The qROF decision matrix is presented in Table 8.

For example:

$$\begin{array}{l} x_{q11}=qROFEWA\;(\partial^{1}{{}_{q}}_{11},\partial^{2}{{}_{q}}_{11},\partial^{3}{{}_{q}}_{11})\\ =\langle \begin{array}{l} {\left(\frac{\prod_{t=1}^{3}{\left(1+\mu_{11t}^{q}\right)}^{\omega_{t}}-\prod_{t=1}^{3}{\left(1-\mu_{11t}^{q}\right)}^{\omega_{t}}}{\prod_{t=1}^{3}{\left(1+\mu_{11t}^{q}\right)}^{\omega_{t}}+\prod_{t=1}^{3}{\left(1-\mu_{11t}^{q}\right)}^{{\overset{-}{\varpi }}_{t}}}\right)}^{\frac{1}{q}}\\ {\left(\frac{2\prod_{t=1}^{3}\vartheta_{11t}^{\omega_{t}q}}{\prod_{t=1}^{3}{\left(2-\vartheta_{ijt}^{q}\right)}^{\omega_{t}}+\prod_{t=1}^{3}\vartheta_{11t}^{\omega_{t}q}}\right)}^{\frac{1}{q}} \end{array}\rangle \end{array}$$

We considered all of the experts to have equal importance. Therefore, $\omega_{1}=\omega_{2}=\omega_{3}=\frac{1}{3}$. In addition, q=3 was also set to formulate the qROF decision matrix (Table 8). Then, the score values of all elements of the qROF decision matrix were calculated using Equation (13).

For example:

$$\begin{array}{l} x_{11}=\frac{\left(\mu_{11}{}^{q}-2\vartheta_{11}{}^{q}-1\right)}{3}+\frac{\lambda }{3}(\mu_{11}{}^{q}+\vartheta_{11}{}^{q}+2)\\ =\frac{\left(0.3167^{3}-2\times 0.7414^{3}-1\right)}{3}+\frac{0.8}{3}(0.3167^{3}+0.7414^{3}+2)=0.0561 \end{array}$$

Here, λ is a constant scalar value. We assumed λ=0.8, as used in [54].

Table 9 represents the score-value-based decision matrix for comparison of the EVs.

Following similar calculations demonstrated in Section 3.1, the final appraisal scores of the EVs were obtained in order to rank them. Table 10 provides the criteria weights, and Table 11 presents the final rankings of the EVs.

Assuming τ=0.5.

The final rankings of the EVs (considering both the technical performance and user opinions) were obtained using the following relation:

(14) $$S_{i(final)}=\frac{\zeta S_{i(technical)}+(1-\zeta )S_{i(user)}}{2}$$

where ζ can take any value between 0 and 1 depending on the choice of the decision makers. However, in this study we assumed that ζ=0.5, because the weights obtained by subjective and objective means were equally appreciated. Table 12 provides the final aggregated rankings of the EVs.

We also examined the consistency among the rankings of the EVs based on the technical attributes and user opinions using the Spearman’s rank correlation (SRC) test (see Table 13). The SRC test is a nonparametric counterpart of the Pearson’s product moment correlation test. The SRC test is performed in cases where the distribution is non-normal, one of the variables is discrete, or the variables are measured on the ordinal scale [55]. In this case, a comparison was made of the orders of the rankings provided by the different MCDM models and between the attractiveness of the EVs based on the technical attributes and user opinions. Hence, it was justified to use the SRC test for evaluating the comparability of the various models. The general definition of the coefficient of the SRC (ρ) is given by:

(15) $$\rho =1-\frac{6\sum d_{i}^{2}}{m(m^{2}-1)}$$

where $d_{i}$ is the difference between two ranks for the $i^{th}$ observation (i.e., a particular alternative in this study), and m is the number of observations.

It can be observed from Table 13 that there is a significant consistency of moderate strength between the rankings based on the technical attributes and user opinions.

### 3.3. Comparative Analysis with Other MCDM Models

To validate the results obtained using the entropy-AROMAN framework, a comparison was made with other the MCDM models, such as the multi-attributive border approximation area comparison (MABAC) [56], proximity indexed value (PIV) [57], and compromise ranking of alternatives from distance to ideal solution (CRADIS) [58] methods.

From Table 14, it can be seen that the results of the rankings with the AROMAN method do not differ significantly from the other models. Therefore, it can be contended that the AROMAN is a considerably reliable solution. The AROMAN method provides the following advantages compared with the MABAC, PIV, and CRADIS methods. AROMAN uses two different normalization schemes: linear max–min (LMM) and vector normalization (VN). As a result, it combats the effect of variations in the performance values (in the decision matrix) on the final order. Previous studies (for instance, [59,60,61]) required the selection of appropriate schemes for normalization and advocated for the use of a combination of normalization techniques instead of using a predefined approach. This helps the decision maker select the best possible solution. LMM is particularly advantageous for comparing the alternatives depending on their closeness to the target reference values (ideal or nonideal) and works well when negative values are present in the decision matrix. However, LMM does not capture the size effect of the criteria units, i.e., differences in the discrete degrees of the performance values of the alternatives under the effect of the criteria. VN is a computationally efficient and symmetric approach that helps in capturing the size effect. Hence, the advantages of AROMAN are reflected in more stable decision making, because the application of two normalizations results in stability in the final order of the alternatives. This is because the different normalizations lead to different final orders. The use of two normalizations contributes to equalizing the value of the alternatives, and based on this, any method can be used without the order of the alternatives remaining stable. Furthermore, AROMAN provides decision makers with flexibility on using thrust on a specific normalization scheme depending on the structure of the decision matrix and requirements of the given problem. In addition, it also separately calculates the sum of the weighted performance values for beneficial and nonbeneficial criteria and provides flexibility in the selection of the coefficient value for the derivation of the final appraisal score.

### 3.4. Sensitivity Analysis

**Figure 2:**
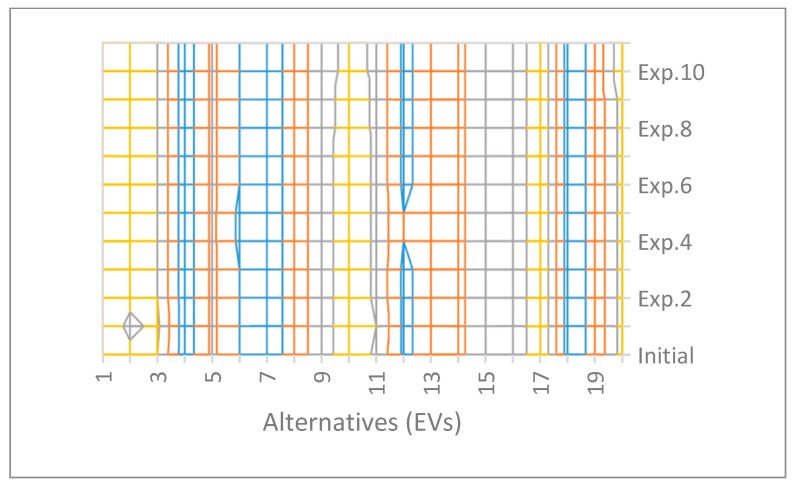
Results of the SA.

The outcomes of MCDM models often become unstable because of sudden changes in the governing conditions, such as changes in the criteria set, variations in the weights, selection of the alternatives, and addition/removal of elements of the decision matrix [62,63,64,65,66,67,68]. Therefore, it is important to carry out a sensitivity analysis (SA) to examine the stability of the solution. The present work follows the SA scheme adopted in [27]. This work includes a good number of external parameters. A sample demonstration of the SA for the user-opinion-based rankings result is provided below. Table 15 shows the SA scheme, and the results of the SA (i.e., the rankings of the EVs under various experimental cases) are also pictorially represented in Figure 2.

Figure 2 shows that most of the alternatives did not change their ranking positions irrespective of the changes in the values of the parameters. The top three and bottom three positions remained unaltered, which allows for the clear differentiation of the performers and nonperformers under all conditions for effective decision making. It can be observed that A2 and A3 showed sensitivity to the changes in the q-values, as they changed their positions. It can be observed that for the ordinary fuzzy sets (q = 1), A2 and A3 did not hold their initial positions. Given the changes in λ, there was a slight variation in the rankings order for A6 and A12, suggesting a change in the preferential orders of A6 and A12. On the other hand, A10 was only susceptible to changes in the value of τ. This means that for any alteration to the relative importance of the beneficial or nonbeneficial criteria effects, A10 showed a slightly better performance. It could also be observed that with variations in the value of ζ, the alternative A20 showed a minor variation in its ranking. However, all variations showed only minor changes in their ranking positions (one or two positions) which reflects the considerable stability of the results.

## 4. Discussion

In order for transport to be sustainable, it is necessary to choose those means of transport that do not harm the environment. EVs represent an alternative to classic transport, because they do not emit harmful gases into the atmosphere, and they contribute to the preservation of the environment [69]. In addition, these vehicles contribute to a reduction in costs, especially transport in urban areas [70]. This is because fossil fuel cars consume more in urban areas and pollute the environment more [71]. However, in addition to these positive aspects, EVs also have negative aspects, namely, car range, charging time, higher cost, and greater vehicle weight [72], while the increase in the number of EVs in the country increases the cost of electricity. Therefore, when choosing an EV, the technical characteristics of these vehicles must be taken into account.

From the analysis of technical performance, it was observed that battery, range, safety, and comfort took priority. However, we noticed some similarities when taking into consideration expert opinions related to users. We observed that cost, electricity consumption, features, aesthetics, and brand image were also priorities. These results support the views in [10,73,74,75]. We further noticed that technical-attribute-based rankings significantly maintained moderate consistency with user-opinion-based rankings. However, the final rankings were more related to the user opinions. Hence, it may be inferred that user opinions influence the choice of EVs. Technical attributes may further reinforce the purchase decision. From the overall rankings of the EVs, it was also noticed that the price of the car was not a top influence. In this regard, the present work adds value to the extant literature. Further, the results of the proposed model showed stability and robustness, as was evident from the validation test and sensitivity analysis. While our approach has many advantages, there are some disadvantages too. Our model poses a slightly higher computational complexity, as it involves hybridization.

The present work contributes to the growing strand of literature in the following ways. First, it provides an apparently rare integrated framework (based on technical attributes and user opinions) to compare popular EVs in India. Secondly, a new extension of the entropy method using two-step normalization with full consistency and qROFN is provided. Hybridization of the entropy method with FUCOM while using double normalizations has not been used in previous research. Third, a novel extension of the very recently developed AROMAN model with qROFN is formulated. Fourth, the current work is the first of its kind that uses a new hybrid entropy AROMAN framework with qROFN using the Einstein aggregation operator.

Our work has a number of technical and managerial implications. First, the present work sheds light on a user-opinion-based selection framework for comparing EVs that may help designers to focus on issues of priority. Secondly, the results may help decision makers formulate strategically appropriate marketing materials. Thirdly, the EA-based qROF entropy with full consistency and two-step normalization with AROMAN can help analysts solve real-life complex issues involving group decision making.

This work posits a number of future scopes of research. First, ongoing work may add further theoretical foundations for technological acceptance and user opinions to conduct a large-scale holistic comparison of EVs. Secondly, it may be interesting to compare the models to various EVs and then examine the commonalities and differences. Thirdly, based on user opinions, a comparison of EVs and the leading normal vehicles could be compared. Fourth, an in-depth exploratory study could be carried out to curate the opinions of users to compare the EVs before applying the MCDM. Fifth, the entropy–AR–MAN framework could be extended using other aggregation techniques (for example, Dombi) and/or other fuzzy numbers for application in real-life decision-making problems. Only a handful of experts (three) participated in this work. As a general scope, future work could include more experts to formulate a focus group discussion and subsequent model building.

## 5. Conclusions

Transportation is an integral part of all aspects of human life. Social well-being and trade and business largely depend on transportation systems. Over the last few decades, global warming has been a top priority for people, organizations, and national leaders. To safeguard lives and livelihoods, it is imperative to reduce carbon emissions and the greenhouse effect. Quite understandably, sustainable city planning to achieve net zero emissions is emphasized by countries (especially those experiencing rapid industrialization and urbanization) across the globe as a long-term strategic action goal. In this regard, transportation is a major area of focus, as it contributes significantly to total carbon emissions. To reduce the CO_2_ footprint for ensuring better air quality, electric cars (ECs) have emerged as a future alternative for sustainable transportation planning. Designing EVs is, today, a distinguished area. ECs are environmentally friendly, as they emit less CO_2_ and other toxic gases and do not use fossil fuels. To this end, the present paper applied a multicriteria decision-making (MCDM)-based framework for the comparison of the leading electric vehicles (EVs) used in India. The comparison was conducted using two dimensions: technical attributes and user opinions. Finally, the outcomes of both of these dimensions were combined to obtain the final rankings. The objective-information-based analysis was carried out for the technical performance analysis based on 12 max-type and one min-type criteria. In this regard, the present work extended the entropy method with two-step normalization and full consistency and used the same for the first time in combination with a very recently developed MCDM model: AROMAN. For the user-opinion-based analysis, qROFNs were used to extend the entropy method with an EA application. It was found that A7, A4, and A18 remained in the top three positions, while A20, A1, and A7 held the bottom three positions irrespective of the changes in the given conditions. It was also observed that A2 and A3 showed sensitivity to changes in the positions of the q-values. With changes in λ, there were slight variations in the order of the rankings for A6 and A12. A10 was only susceptible to changes in the value of τ. It was seen that with variation in the value of ζ, the alternative A20 showed a minor variation in its ranking. Further, the results of the rankings with AROMAN compared with the other MCDM models were found to be consistent. Hence, the results of the proposed model showed stability and robustness, as evident from the validation test and sensitivity analysis. The technical-attribute-based rankings significantly maintained a moderate consistency with the user-opinion-based rankings. However, the final rankings were more related to the user opinions. Hence, it may be inferred that user opinions influence the choice of EVs.

## Figures and Tables

**Table 1:** List of technical attributes (i.e., criteria) for comparison of the EVs.

S/L	Technical Attribute	UOM	Effect Direction	References
TA1	Max Torque	Newton-Meters (Nm) @rpm	(+)	[8,15,17]
TA2	Max Power	Brake Horsepower (bhp) @rpm	(+)	[15,17,51]
TA3	Boot Space	(Liters)	(+)	[14,17]
TA4	Battery Capacity	(KWh)	(+)	[12,17,19]
TA5	Range	(km)	(+)	[10,14,17,25]
TA6	Acceleration	(sec)	(+)	[12,17,25]
TA7	Wheel Base	(mm)	(+)	[18,52]
TA8	Wheel Size	(Inch)	(+)	[14,52]
TA9	No. of Airbags	No	(+)	[14,22]
TA10	Battery Warranty	(Years)	(+)	[18,51]
TA11	Seating Capacity	No	(+)	[15,17]
TA12	No. of Doors	No	(+)	[51,52,53]
TA13	Price (Cr)	Rs. Cr.	(−)	[12,14,17]

**Table 2:** List of factors (i.e., criteria) that influence customers’ choice of EV.

S/L	User-Centric Criteria	Effect Direction	References
UA1	Mileage	(+)	[14]
UA2	User Friendliness of the Technology	(+)	[50]
UA3	Compatibility with other Technologies	(+)	[15]
UA4	Features	(+)	[14,22]
UA5	Comfort during the Ride	(+)	[50]
UA6	Aesthetics	(+)	[14,15]
UA7	Brand Image	(+)	[15]
UA8	Ease of Maintenance	(+)	[19]
UA9	After Sales Support	(+)	[50]
UA10	Safety	(+)	[16]
UA11	Environment Friendliness	(+)	[15,16]
UA12	Electricity Consumption	(−)	[16,26]
UA13	Costing	(−)	[16,17,19,22]

**Table 3:** Five-point qROF linguistic scale.

Linguistic Description	Code	*µ*	*ϑ*
Very Low	VL	0.25	0.85
Low	L	0.40	0.70
Moderate	M	0.55	0.55
High	H	0.70	0.40
Very High	VH	0.85	0.25

**Table 4:** Normalized decision matrix (technical attributes).

Model	TA1	TA2	TA3	TA4	TA5	TA6	TA7	TA8	TA9	TA10	TA11	TA12	TA13
A1	0.0182	0.0300	0.0432	0.0432	0.1580	0.1242	0.1696	0.0994	0.0497	0.3085	0.3077	0.3094	0.2483
A2	0.0687	0.0637	0.1168	0.1006	0.2432	0.2566	0.1874	0.1612	0.0497	0.3085	0.3077	0.3094	0.2417
A3	0.0668	0.0567	0.1168	0.1006	0.1559	0.2927	0.1874	0.1612	0.0497	0.3085	0.3077	0.3094	0.2439
A4	0.3547	0.3688	0.2172	0.3350	0.3243	0.0320	0.3163	0.2539	0.3328	0.3085	0.3077	0.3094	0.1095
A5	0.2210	0.1944	0.1596	0.2312	0.2300	0.0921	0.0341	0.2539	0.2519	0.3085	0.3077	0.3094	0.2149
A6	0.2006	0.1535	0.2654	0.2292	0.2872	0.0360	0.2602	0.2539	0.2924	0.3085	0.3077	0.3094	0.2101
A7	0.1356	0.1608	0.1971	0.2518	0.3162	0.1242	0.2522	0.2539	0.2924	0.3085	0.3077	0.2142	0.2046
A8	0.2173	0.2346	0.1811	0.2849	0.2314	0.0600	0.2602	0.3157	0.2924	0.3085	0.3077	0.3094	0.1142
A9	0.0798	0.0801	0.1824	0.1348	0.2600	0.2366	0.2024	0.1921	0.2115	0.3085	0.3077	0.3094	0.2378
A10	0.0761	0.0837	0.0238	0.0731	0.1266	0.1884	0.1868	0.1921	0.0901	0.3085	0.2128	0.3094	0.2185
A11	0.2344	0.1904	0.3216	0.2730	0.2663	0.0881	0.2765	0.2230	0.2115	0.3085	0.3077	0.3094	0.1665
A12	0.2099	0.1541	0.2172	0.2069	0.2163	0.1403	0.2783	0.3157	0.2115	0.3085	0.3077	0.3094	0.1709
A13	0.0427	0.0401	0.2708	0.2093	0.2279	0.2165	0.2421	0.1921	0.1306	0.3085	0.3077	0.3094	0.2344
A14	0.2842	0.3118	0.3243	0.2849	0.2460	0.0280	0.2607	0.2848	0.2115	0.3085	0.3077	0.2142	0.1190
A15	0.2099	0.2547	0.3243	0.2849	0.2481	0.0600	0.2607	0.2848	0.2519	0.3085	0.3077	0.2142	0.1329
A16	0.2225	0.1432	0.3243	0.2905	0.2324	0.1242	0.2653	0.2848	0.2924	0.3085	0.3077	0.3094	0.1746
A17	0.0092	0.0032	0.0833	0.0118	0.0777	0.1443	0.0993	0.0376	0.0497	0.0219	0.0231	0.0238	0.2525
A18	0.2582	0.1944	0.2172	0.2382	0.2614	0.1002	0.2553	0.2230	0.3328	0.3085	0.3077	0.3094	0.1830
A19	0.1226	0.0602	0.2427	0.0961	0.2537	0.2125	0.2059	0.1921	0.2115	0.3085	0.3077	0.3094	0.2382
A20	0.0096	0.0139	0.2239	0.0467	0.0148	0.3449	0.2113	0.0994	0.0092	0.0219	0.3077	0.2142	0.2490

**Table 5:** Weights of the technical attributes.

Criterion	*E_j_*	*φ* (*k*/*k* + 1)	*w* (*k*/*k* + 1)	*w* (*k*/*k* + 2)	*w*
TA11	2.3180	1.0132	1.0132	1.0367	0.08914
TA12	2.2877	1.0232	1.0232	1.0655	0.08798
TA10	2.2358	1.0413	1.0413	1.0613	0.08598
TA7	2.1472	1.0192	1.0192	1.0209	0.08257
TA5	2.1067	1.0016	1.0016	1.0029	0.08101
TA8	2.1033	1.0013	1.0013	1.0491	0.08088
TA13	2.1007	1.0478	1.0478	1.1135	0.08078
TA3	2.0048	1.0627	1.0627	1.0668	0.07709
TA4	1.8865	1.0038	1.0038	1.1179	0.07254
TA9	1.8794	1.1136	1.1136	1.1310	0.07227
TA6	1.6876	1.0156	1.0156	1.0645	0.06489
TA1	1.6617	1.0482	1.0482		0.06390
TA2	1.5853				0.06096
	DFC	0.00002		Σ	1.0000

**Table 6:** Weighted normalized decision matrix (technical attributes).

S/L	TA1	TA2	TA3	TA4	TA5	TA6	TA7	TA8	TA9	TA10	TA11	TA12	TA13
A1	0.0012	0.0018	0.0033	0.0031	0.0128	0.0081	0.0140	0.0080	0.0036	0.0265	0.0274	0.0272	0.0201
A2	0.0044	0.0039	0.0090	0.0073	0.0197	0.0167	0.0155	0.0130	0.0036	0.0265	0.0274	0.0272	0.0195
A3	0.0043	0.0035	0.0090	0.0073	0.0126	0.0190	0.0155	0.0130	0.0036	0.0265	0.0274	0.0272	0.0197
A4	0.0227	0.0225	0.0167	0.0243	0.0263	0.0021	0.0261	0.0205	0.0241	0.0265	0.0274	0.0272	0.0088
A5	0.0141	0.0118	0.0123	0.0168	0.0186	0.0060	0.0028	0.0205	0.0182	0.0265	0.0274	0.0272	0.0174
A6	0.0128	0.0094	0.0205	0.0166	0.0233	0.0023	0.0215	0.0205	0.0211	0.0265	0.0274	0.0272	0.0170
A7	0.0087	0.0098	0.0152	0.0183	0.0256	0.0081	0.0208	0.0205	0.0211	0.0265	0.0274	0.0188	0.0165
A8	0.0139	0.0143	0.0140	0.0207	0.0187	0.0039	0.0215	0.0255	0.0211	0.0265	0.0274	0.0272	0.0092
A9	0.0051	0.0049	0.0141	0.0098	0.0211	0.0154	0.0167	0.0155	0.0153	0.0265	0.0274	0.0272	0.0192
A10	0.0049	0.0051	0.0018	0.0053	0.0103	0.0122	0.0154	0.0155	0.0065	0.0265	0.0190	0.0272	0.0177
A11	0.0150	0.0116	0.0248	0.0198	0.0216	0.0057	0.0228	0.0180	0.0153	0.0265	0.0274	0.0272	0.0135
A12	0.0134	0.0094	0.0167	0.0150	0.0175	0.0091	0.0230	0.0255	0.0153	0.0265	0.0274	0.0272	0.0138
A13	0.0027	0.0024	0.0209	0.0152	0.0185	0.0141	0.0200	0.0155	0.0094	0.0265	0.0274	0.0272	0.0189
A14	0.0182	0.0190	0.0250	0.0207	0.0199	0.0018	0.0215	0.0230	0.0153	0.0265	0.0274	0.0188	0.0096
A15	0.0134	0.0155	0.0250	0.0207	0.0201	0.0039	0.0215	0.0230	0.0182	0.0265	0.0274	0.0188	0.0107
A16	0.0142	0.0087	0.0250	0.0211	0.0188	0.0081	0.0219	0.0230	0.0211	0.0265	0.0274	0.0272	0.0141
A17	0.0006	0.0002	0.0064	0.0009	0.0063	0.0094	0.0082	0.0030	0.0036	0.0019	0.0021	0.0021	0.0204
A18	0.0165	0.0118	0.0167	0.0173	0.0212	0.0065	0.0211	0.0180	0.0241	0.0265	0.0274	0.0272	0.0148
A19	0.0078	0.0037	0.0187	0.0070	0.0206	0.0138	0.0170	0.0155	0.0153	0.0265	0.0274	0.0272	0.0192
A20	0.0006	0.0008	0.0173	0.0034	0.0012	0.0224	0.0174	0.0080	0.0007	0.0019	0.0274	0.0188	0.0201

**Table 7:** Ranking of the EVs (technical attributes).

Model	*P_i_*	*L_i_*	*S_i_*	Rank	Model	*P_i_*	*L_i_*	*S_i_*	Rank
A1	0.1371	0.0201	0.5119	18	A11	0.2358	0.0135	0.6016	5
A2	0.1742	0.0195	0.5571	15	A12	0.2262	0.0138	0.5931	7
A3	0.1689	0.0197	0.5514	16	A13	0.1999	0.0189	0.5847	11
A4	0.2664	0.0088	0.6102	2	A14	0.2372	0.0096	0.5851	10
A5	0.2024	0.0174	0.5816	13	A15	0.2342	0.0107	0.5875	8
A6	0.2292	0.0170	0.6090	3	A16	0.2431	0.0141	0.6118	1
A7	0.2209	0.0165	0.5986	6	A17	0.0446	0.0204	0.3540	20
A8	0.2348	0.0092	0.5806	14	A18	0.2344	0.0148	0.6057	4
A9	0.1989	0.0192	0.5846	12	A19	0.2005	0.0192	0.5865	9
A10	0.1498	0.0177	0.5199	17	A20	0.1200	0.0201	0.4882	19

**Table 8:** qROF decision matrix (aggregated response).

**Model**	**UA1**		**UA2**		**UA3**		**UA4**		**UA5**		**UA6**		**UA7**	
A1	0.3167	0.7414	0.8500	0.2494	0.7000	0.3958	0.5500	0.5355	0.4000	0.6640	0.2500	0.7774	0.7000	0.3958
A2	0.5099	0.5777	0.7000	0.3958	0.5500	0.5355	0.5500	0.5355	0.4000	0.6640	0.4616	0.6208	0.7000	0.3958
A3	0.2500	0.7774	0.7627	0.3402	0.6589	0.4392	0.5500	0.5355	0.5500	0.5355	0.5099	0.5777	0.8500	0.2494
A4	0.8500	0.2494	0.6101	0.4859	0.8115	0.2915	0.7000	0.3958	0.8115	0.2915	0.7000	0.3958	0.8500	0.2494
A5	0.5500	0.5355	0.4000	0.6640	0.4000	0.6640	0.5500	0.5355	0.7871	0.3251	0.4000	0.6640	0.7000	0.3958
A6	0.8500	0.2494	0.4000	0.6640	0.3167	0.7414	0.4000	0.6640	0.6101	0.4859	0.7000	0.3958	0.5500	0.5355
A7	0.8500	0.2494	0.5500	0.5355	0.8115	0.2915	0.8500	0.2494	0.7000	0.3958	0.7000	0.3958	0.8500	0.2494
A8	0.5099	0.5777	0.5500	0.5355	0.7000	0.3958	0.8500	0.2494	0.8500	0.2494	0.6589	0.4392	0.8500	0.2494
A9	0.6101	0.4859	0.2500	0.7774	0.4000	0.6640	0.5500	0.5355	0.5500	0.5355	0.6101	0.4859	0.5099	0.5777
A10	0.2500	0.7774	0.2500	0.7774	0.3167	0.7414	0.5500	0.5355	0.5500	0.5355	0.4000	0.6640	0.6101	0.4859
A11	0.7000	0.3958	0.7000	0.3958	0.7627	0.3402	0.5500	0.5355	0.7000	0.3958	0.5500	0.5355	0.8500	0.2494
A12	0.5099	0.5777	0.8500	0.2494	0.8115	0.2915	0.7000	0.3958	0.8500	0.2494	0.7000	0.3958	0.7000	0.3958
A13	0.4616	0.6208	0.7000	0.3958	0.7000	0.3958	0.5500	0.5355	0.7000	0.3958	0.7000	0.3958	0.4000	0.6640
A14	0.7000	0.3958	0.5500	0.5355	0.5500	0.5355	0.7000	0.3958	0.7000	0.3958	0.7000	0.3958	0.8500	0.2494
A15	0.7000	0.3958	0.7000	0.3958	0.5500	0.5355	0.7000	0.3958	0.7000	0.3958	0.7000	0.3958	0.7000	0.3958
A16	0.6101	0.4859	0.4000	0.6640	0.4000	0.6640	0.7000	0.3958	0.4000	0.6640	0.7627	0.3402	0.7000	0.3958
A17	0.2500	0.7774	0.3167	0.7414	0.5500	0.5355	0.5500	0.5355	0.2500	0.7774	0.5099	0.5777	0.5500	0.5355
A18	0.7000	0.3958	0.5099	0.5777	0.8500	0.2494	0.7000	0.3958	0.7000	0.3958	0.8500	0.2494	0.8500	0.2494
A19	0.7000	0.3958	0.8500	0.2494	0.7000	0.3958	0.2500	0.7774	0.2500	0.7774	0.4000	0.6640	0.7000	0.3958
A20	0.2500	0.7774	0.7000	0.3958	0.5500	0.5355	0.2500	0.7774	0.5500	0.5355	0.5500	0.5355	0.7000	0.3958
**Model**	**UA8**		**UA9**		**UA10**		**UA11**		**UA12**		**UA13**			
A1	0.2500	0.7774	0.4000	0.6640	0.2500	0.7774	0.5500	0.5355	0.2500	0.7774	0.2500	0.7774		
A2	0.2500	0.7774	0.4616	0.6208	0.4000	0.6640	0.5099	0.5777	0.2500	0.7774	0.4000	0.6640		
A3	0.4000	0.6640	0.5099	0.5777	0.2500	0.7774	0.5500	0.5355	0.2500	0.7774	0.5434	0.5682		
A4	0.3167	0.7414	0.4000	0.6640	0.8115	0.2915	0.7000	0.3958	0.5500	0.5355	0.8115	0.2915		
A5	0.6589	0.4392	0.7000	0.3958	0.7000	0.3958	0.4000	0.6640	0.4000	0.6640	0.4000	0.6640		
A6	0.7627	0.3402	0.7000	0.3958	0.7000	0.3958	0.6101	0.4859	0.5500	0.5355	0.4000	0.6640		
A7	0.5500	0.5355	0.5500	0.5355	0.7000	0.3958	0.5500	0.5355	0.7000	0.3958	0.5099	0.5777		
A8	0.2500	0.7774	0.3167	0.7414	0.7000	0.3958	0.7000	0.3958	0.5500	0.5355	0.8115	0.2915		
A9	0.7000	0.3958	0.5099	0.5777	0.5500	0.5355	0.8500	0.2494	0.4000	0.6640	0.2500	0.7774		
A10	0.7000	0.3958	0.6101	0.4859	0.4616	0.6208	0.7000	0.3958	0.4000	0.6640	0.4000	0.6640		
A11	0.2500	0.7774	0.2500	0.7774	0.5099	0.5777	0.4000	0.6640	0.8500	0.2494	0.7000	0.3958		
A12	0.5099	0.5777	0.5500	0.5355	0.5500	0.5355	0.5500	0.5355	0.5500	0.5355	0.7000	0.3958		
A13	0.6101	0.4859	0.7000	0.3958	0.4000	0.6640	0.7000	0.3958	0.4616	0.6208	0.2500	0.7774		
A14	0.5500	0.5355	0.5500	0.5355	0.5500	0.5355	0.5500	0.5355	0.5099	0.5777	0.8500	0.2494		
A15	0.4000	0.6640	0.5500	0.5355	0.7000	0.3958	0.2500	0.7774	0.6101	0.4859	0.8500	0.2494		
A16	0.5500	0.5355	0.5500	0.5355	0.7627	0.3402	0.4000	0.6640	0.5500	0.5355	0.7000	0.3958		
A17	0.5500	0.5355	0.7000	0.3958	0.2500	0.7774	0.5500	0.5355	0.4000	0.6640	0.2500	0.7774		
A18	0.2500	0.7774	0.2500	0.7774	0.8500	0.2494	0.7627	0.3402	0.5500	0.5355	0.7627	0.3402		
A19	0.8500	0.2494	0.8500	0.2494	0.5500	0.5355	0.3167	0.7414	0.2500	0.7774	0.4616	0.6208		
A20	0.8500	0.2494	0.8500	0.2494	0.2500	0.7774	0.4000	0.6640	0.2500	0.7774	0.2500	0.7774		

**Table 9:** Score-value-based decision matrix (user opinions).

Model	UA1	UA2	UA3	UA4	UA5	UA6	UA7	UA8	UA9	UA10	UA11	UA12	UA13
A1	0.0561	0.5623	0.3810	0.2384	0.1213	0.0214	0.3810	0.0214	0.1213	0.0214	0.2384	0.0214	0.0214
A2	0.2024	0.3810	0.2384	0.2384	0.1213	0.1633	0.3810	0.0214	0.1633	0.1213	0.2024	0.0214	0.1213
A3	0.0214	0.4505	0.3378	0.2384	0.2384	0.2024	0.5623	0.1213	0.2024	0.0214	0.2384	0.0214	0.2229
A4	0.5623	0.2904	0.5107	0.3810	0.5107	0.3810	0.5623	0.0561	0.1213	0.5107	0.3810	0.2384	0.5107
A5	0.2384	0.1213	0.1213	0.2384	0.4789	0.1213	0.3810	0.3378	0.3810	0.3810	0.1213	0.1213	0.1213
A6	0.5623	0.1213	0.0561	0.1213	0.2904	0.3810	0.2384	0.4505	0.3810	0.3810	0.2904	0.2384	0.1213
A7	0.5623	0.2384	0.5107	0.5623	0.3810	0.3810	0.5623	0.2384	0.2384	0.3810	0.2384	0.3810	0.2024
A8	0.2024	0.2384	0.3810	0.5623	0.5623	0.3378	0.5623	0.0214	0.0561	0.3810	0.3810	0.2384	0.5107
A9	0.2904	0.0214	0.1213	0.2384	0.2384	0.2904	0.2024	0.3810	0.2024	0.2384	0.5623	0.1213	0.0214
A10	0.0214	0.0214	0.0561	0.2384	0.2384	0.1213	0.2904	0.3810	0.2904	0.1633	0.3810	0.1213	0.1213
A11	0.3810	0.3810	0.4505	0.2384	0.3810	0.2384	0.5623	0.0214	0.0214	0.2024	0.1213	0.5623	0.3810
A12	0.2024	0.5623	0.5107	0.3810	0.5623	0.3810	0.3810	0.2024	0.2384	0.2384	0.2384	0.2384	0.3810
A13	0.1633	0.3810	0.3810	0.2384	0.3810	0.3810	0.1213	0.2904	0.3810	0.1213	0.3810	0.1633	0.0214
A14	0.3810	0.2384	0.2384	0.3810	0.3810	0.3810	0.5623	0.2384	0.2384	0.2384	0.2384	0.2024	0.5623
A15	0.3810	0.3810	0.2384	0.3810	0.3810	0.3810	0.3810	0.1213	0.2384	0.3810	0.0214	0.2904	0.5623
A16	0.2904	0.1213	0.1213	0.3810	0.1213	0.4505	0.3810	0.2384	0.2384	0.4505	0.1213	0.2384	0.3810
A17	0.0214	0.0561	0.2384	0.2384	0.0214	0.2024	0.2384	0.2384	0.3810	0.0214	0.2384	0.1213	0.0214
A18	0.3810	0.2024	0.5623	0.3810	0.3810	0.5623	0.5623	0.0214	0.0214	0.5623	0.4505	0.2384	0.4505
A19	0.3810	0.5623	0.3810	0.0214	0.0214	0.1213	0.3810	0.5623	0.5623	0.2384	0.0561	0.0214	0.1633
A20	0.0214	0.3810	0.2384	0.0214	0.2384	0.2384	0.3810	0.5623	0.5623	0.0214	0.1213	0.0214	0.0214

**Table 10:** Criteria weights (user opinions).

Criteria	*E_j_*	*φ* (*k*/*k* + 1)	*w* (*k*/*k* + 1)	*w* (*k*/*k* + 2)	*w*
UA12	2.2146	1.0806	1.0806	1.0881	0.0944
UA13	2.0494	1.0069	1.0069	1.0993	0.0873
UA7	2.0353	1.0917	1.0917	1.1105	0.0867
UA6	1.8643	1.0172	1.0172	1.0235	0.0794
UA4	1.8328	1.0062	1.0062	1.0294	0.0781
UA5	1.8215	1.0230	1.0230	1.0453	0.0776
UA3	1.7805	1.0217	1.0217	1.0252	0.0759
UA11	1.7427	1.0034	1.0034	1.0305	0.0743
UA2	1.7367	1.0270	1.0270	1.0734	0.0740
UA9	1.6910	1.0452	1.0452	1.0455	0.0721
UA10	1.6180	1.0003	1.0003	1.1046	0.0689
UA1	1.6174	1.1042	1.1042		0.0689
UA8	1.4648				0.0624
	DFC	0.00003		**Σ**	1.0000

**Table 11:** Rankings of the EVs (user opinions).

Model	*P_i_*	*L_i_*	*S_i_*	Rank	Model	*P_i_*	*L_i_*	*S_i_*	Rank
A1	0.0942	0.0463	0.5220	17	A11	0.1357	0.0264	0.5309	16
A2	0.0970	0.0438	0.5206	18	A12	0.1765	0.0330	0.6016	4
A3	0.1191	0.0413	0.5482	13	A13	0.1403	0.0434	0.5829	7
A4	0.1957	0.0297	0.6148	2	A14	0.1593	0.0292	0.5699	9
A5	0.1267	0.0418	0.5602	11	A15	0.1459	0.0274	0.5475	14
A6	0.1401	0.0394	0.5727	8	A16	0.1277	0.0330	0.5389	15
A7	0.1962	0.0345	0.6287	1	A17	0.0788	0.0442	0.4911	20
A8	0.1701	0.0297	0.5848	5	A18	0.1887	0.0312	0.6111	3
A9	0.1189	0.0442	0.5552	12	A19	0.1416	0.0427	0.5831	6
A10	0.0926	0.0418	0.5086	19	A20	0.1203	0.0463	0.5619	10

**Table 12:** Aggregated final rankings of the EVs (technical performance and user opinions).

Model	*S_i_*				Model	*S_i_*			
	Technical	User	Final	Rank		Technical	User	Final	Rank
A1	0.5119	0.5220	0.2585	18	A11	0.6016	0.5309	0.2831	14
A2	0.5571	0.5206	0.2694	16	A12	0.5931	0.6016	0.2987	4
A3	0.5514	0.5482	0.2749	15	A13	0.5847	0.5829	0.2919	7
A4	0.6102	0.6148	0.3062	2	A14	0.5851	0.5699	0.2888	9
A5	0.5816	0.5602	0.2855	11	A15	0.5875	0.5475	0.2838	13
A6	0.6090	0.5727	0.2954	5	A16	0.6118	0.5389	0.2877	10
A7	0.5986	0.6287	0.3068	1	A17	0.3540	0.4911	0.2113	20
A8	0.5806	0.5848	0.2913	8	A18	0.6057	0.6111	0.3042	3
A9	0.5846	0.5552	0.2850	12	A19	0.5865	0.5831	0.2924	6
A10	0.5199	0.5086	0.2571	19	A20	0.4882	0.5619	0.2625	17

**Table 13:** SRC test-I.

	Method	
Coefficient		Rank (User Opinions)
Spearman’s rho	Rank (Technical Attributes)	0.528 *
Sig. (2-tailed)		0.017

* Correlation is significant at the 0.05 level (2-tailed).

**Table 14:** SRC test-II.

	Method			
Coefficient		MABAC	PIV	CRADIS
Spearman’s rho	AROMAN	0.928 **	0.905 **	0.926 **

** Correlation is significant at the 0.01 level (2-tailed).

**Table 15:** Experimental results of the SA.

	Parameter Values				
Case	*q*	*λ*	*β*	*τ*	*ζ*
Initial	3	0.8	0.5	0.5	0.5
Exp. 1	1	0.8	0.5	0.5	0.5
Exp. 2	5	0.8	0.5	0.5	0.5
Exp. 3	10	0.8	0.5	0.5	0.5
Exp. 4	3	0.9	0.5	0.5	0.5
Exp. 5	3	0.4	0.5	0.5	0.5
Exp. 6	3	0.8	0.3	0.5	0.5
Exp. 7	3	0.8	0.8	0.5	0.5
Exp. 8	3	0.8	0.5	0.9	0.5
Exp. 9	3	0.8	0.5	0.2	0.5
Exp. 10	3	0.8	0.5	0.5	0.4
Exp. 11	3	0.8	0.5	0.5	0.8

## Data Availability

Supporting information is provided. Further information is available upon request.

## Appendix A. Preliminaries of qROFS

In this section, some preliminary concepts and operations of the qROFS are described briefly [30].

**Definition A1.** 
*A q-ROFS is defined as:*

(A1)
$${\tilde{A}}_{q}=\left\{\langle x,\mu_{{\tilde{A}}_{q}}(x),\vartheta_{{\tilde{A}}_{q}}(x)\rangle ;x\in Χ\right\}$$

*where Χ is the universe of discourse.*

$\mu_{{\tilde{A}}_{q}}(x):Χ\to [0,1]$ *and $\vartheta_{{\tilde{A}}_{q}}(x):Χ\to [0,1]$ are the degree of membership (DoM) and degree of nonmembership (DoNM), respectively.*

$$0\leq {\left(\mu_{{\tilde{A}}_{q}}(x)\right)}^{q}+{\left(\vartheta_{{\tilde{A}}_{q}}(x)\right)}^{q}\leq 1;\forall x\in Χ$$

*The degree of indeterminacy (DoI) is given by:*

(A2) $$\pi_{{\tilde{A}}_{q}}(x)=\sqrt[{q}]{1-\mu_{{\tilde{A}}_{P}}(x){)}^{q}-{\left(\vartheta_{{\tilde{A}}_{P}}(x)\right)}^{q}}\forall x\in Χ;\pi_{{\tilde{A}}_{q}}(x):Χ\to [0,1]$$

*If q=1: ${\tilde{A}}_{q}$ is the Atanassov’s intuitionistic fuzzy set (IFS);*
*If q=2: ${\tilde{A}}_{q}$ is the Pythagorean fuzzy sets (PyFS);*
*If q=3: ${\tilde{A}}_{q}$ is converted into Fermatean fuzzy sets (FFS).*

*Let $A_{q}=\left\{\mu ,\vartheta \right\}$ be a general qROFN representing the qROFS for convenience in explaining and applying the concepts while keeping the meaning of the terms and their fundamental definitions.*

**Definition A2.** 
*Basic operations of q-ROFN.*

*Let $A_{q}=\left\{\mu ,\vartheta \right\},A_{q1}=\left\{\mu_{1},\vartheta_{1}\right\}$. $A_{q2}=\left\{\mu_{2},\vartheta_{2}\right\}$ are the three q-ROFNs. Then, we have the following operations:*

(A3)
$$A_{q}{}^{c}=\left\{\vartheta ,\mu \right\}$$

(A4)
$$A_{q1}⊕A_{q2}=\left\{\sqrt[{q}]{\mu_{1}^{q}+\mu_{2}^{q}-\mu_{1}^{q}\mu_{2}^{q}},\vartheta_{1}\vartheta_{2}\right\}$$

(A5)
$$A_{q}{}_{1}⊗A_{q}{}_{2}=\left\{\mu_{1}\mu_{2},\sqrt[{q}]{\vartheta_{1}^{q}+\vartheta_{2}^{q}-\vartheta_{1}^{q}\vartheta_{2}^{q}}\right\}$$

(A6)
$$\alpha A_{q}=\left\{\sqrt[{q}]{1-{\left(1-\mu^{q}\right)}^{\alpha }},\vartheta^{\alpha }\right\};\;\alpha \;\mathrm{is a constant}$$

(A7)
$$A_{q}{}^{\alpha }=\left\{\mu^{\alpha },\sqrt[{q}]{1-{\left(1-\vartheta^{q}\right)}^{\alpha }}\right\}$$

**Definition A3.** 
*Score and accuracy function.*

*As per the definition provided by Peng and Dai* [54], *the definition of the score function (SF) is given below:*

(A8) $${ℑ}^{*}=\frac{\left(\mu^{q}-2\vartheta^{q}-1\right)}{3}+\frac{\lambda }{3}(\mu^{q}+\vartheta^{q}+2);\lambda \in [0,1]$$

*Here, λ is a constant scalar value.*

*The accuracy function (AF) is defined by Liu and Wang* [76] *as:*

(A9) $$H=\mu^{q}+\vartheta^{q};H\in [0,1]$$

*The rules for comparison are as follows:*

*If:*

$SF_{1}≻SF_{2}\Rightarrow A_{q}{}_{1}≻A_{q}{}_{2}$ *;*
*Else if $SF_{1}≺SF_{2}\Rightarrow A_{q}{}_{1}≺A_{q}{}_{2}$;*
*Else if $SF_{1}=SF_{2}$ then;*
*If $AF_{1}≻AF_{2}\Rightarrow A_{q}{}_{1}≻A_{q}{}_{2}$.*

**Definition A4.** 
*qROF-weighted averaging operator (q-ROFWA).*

*The definition is provided by Liu and Wang* [76]:

(A10) $$q-ROFWA(A_{q}{}_{1},A_{q}{}_{2},\;\ldots ,\;A_{q}{}_{r})=\langle (1-\prod_{k=1}^{r}{\left(1-\mu_{k}^{q}\right)}^{w_{k}}{)}^{\frac{1}{q}},\prod_{k=1}^{r}\vartheta_{k}^{w_{k}}\rangle$$

*Here, $w_{k}$ is the weight of $A_{q}{}_{k}$*.

**Definition A5.** 
*Einstein sum and product.*

*The definitions, as provided in Weber* [77], *are given below:*

*Let a, b ∈ [0,1]*.

*Then, the Einstein sum and product are derived as:*

(A11)
$$a{⊕}_{\varepsilon }b=\frac{a+b}{1+ab}$$

(A12)
$$a{⊗}_{\varepsilon }b=\frac{ab}{1+(1-a)(1-b)}$$

**Definition A6.** 
*qROF-weighted aggregation.*

*The qROF Einstein-weighted average (qROFEWA) for the qROFNs $A_{q}{}_{1},A_{q}{}_{2},\;\ldots ,\;A_{q}{}_{r}$ is computed* [48] *as:*

(A13) $$\begin{array}{l} qROFEWA\;(A_{q}{}_{1},A_{q}{}_{2},\;\ldots ,\;A_{q}{}_{r})\\ =\langle \begin{array}{l} {\left(\frac{\prod_{i=1}^{r}{\left(1+\mu_{i}^{q}\right)}^{w_{i}}-\prod_{i=1}^{r}{\left(1-\mu_{i}^{q}\right)}^{w_{i}}}{\prod_{i=1}^{r}{\left(1+\mu_{i}^{q}\right)}^{w_{i}}+\prod_{i=1}^{r}{\left(1-\mu_{i}^{q}\right)}^{w_{i}}}\right)}^{\frac{1}{q}}\\ {\left(\frac{2\prod_{i=1}^{r}\vartheta_{i}^{w_{i}q}}{\prod_{i=1}^{r}{\left(2-\vartheta_{i}^{q}\right)}^{w_{i}}+\prod_{i=1}^{r}\vartheta_{i}^{w_{i}q}}\right)}^{\frac{1}{q}} \end{array}\rangle \end{array}$$

*Similarly, the qROF Einstein-weighted geometric average (qROFEWG) for the qROFNs is defined as:*

(A14) $$\begin{array}{l} qROFEWG\;(A_{q}{}_{1},A_{q}{}_{2},\;\ldots ,\;A_{q}{}_{r})\\ =\langle \begin{array}{l} {\left(\frac{2\prod_{i=1}^{r}\mu_{i}^{w_{i}q}}{\prod_{i=1}^{r}{\left(2-\mu_{i}^{q}\right)}^{w_{i}}+\prod_{i=1}^{r}\mu_{i}^{w_{i}q}}\right)}^{\frac{1}{q}}\\ {\left(\frac{\prod_{i=1}^{r}{\left(1+\vartheta_{i}^{q}\right)}^{w_{i}}-\prod_{i=1}^{r}{\left(1-\vartheta_{i}^{q}\right)}^{w_{i}}}{\prod_{i=1}^{r}{\left(1+\vartheta_{i}^{q}\right)}^{w_{i}}+\prod_{i=1}^{r}{\left(1-\vartheta_{i}^{q}\right)}^{w_{i}}}\right)}^{\frac{1}{q}} \end{array}\rangle \end{array}$$

## Appendix B

**Table A1:** Decision matrix for comparing the EVs based on technical attributes.

Model	TA1	TA2	TA3	TA4	TA5	TA6	TA7	TA8	TA9	TA10	TA11	TA12	TA13
A1	114	73.75	240	24	315	5.7	2400	14	2	8	5	5	0.10
A2	250	141	350	40.5	437	9	2498	16	2	8	5	5	0.19
A3	245	127	350	40.5	312	9.9	2498	16	2	8	5	5	0.16
A4	1020	751	500	107.8	553	3.4	3210	19	9	8	5	5	2.00
A5	660	402.3	414	78	418	4.9	1652	19	7	8	5	5	0.56
A6	605	320.6	572	77.4	500	3.5	2900	19	8	8	5	5	0.62
A7	430	335.3	470	83.9	541.5	5.7	2856	19	8	8	5	4	0.70
A8	650	482.8	446	93.4	420	4.1	2900	21	8	8	5	5	1.94
A9	280	173.8	448	50.3	461	8.5	2581	17	6	8	5	5	0.25
A10	270	181	211	32.6	270	7.3	2495	17	3	8	4	5	0.51
A11	696	394.3	656	90	470	4.8	2990	18	6	8	5	5	1.22
A12	630	321.8	500	71	398.5	6.1	3000	21	6	8	5	5	1.16
A13	180	93.87	580	71.7	415	8	2800	17	4	8	5	5	0.29
A14	830	637	660	93.4	441	3.3	2903	20	6	8	5	4	1.87
A15	630	523	660	93.4	444	4.1	2903	20	7	8	5	4	1.68
A16	664	300	660	95	421.5	5.7	2928	20	8	8	5	5	1.11
A17	90	20.11	300	15	200	6.2	2012	12	2	3	2	2	0.05
A18	760	402.3	500	80	463	5.1	2873	18	9	8	5	5	1.00
A19	395	134.1	538	39.2	452	7.9	2600	17	6	8	5	5	0.24
A20	91	41.57	510	25	110	11.2	2630	14	1	3	5	4	0.09

## Appendix C

**Table A2:** Rating of the alternatives by expert 1.

Model	UA1	UA2	UA3	UA4	UA5	UA6	UA7	UA8	UA9	UA10	UA11	UA12	UA13
A1	VL	VH	H	M	L	VL	H	VL	L	VL	M	VL	VL
A2	M	H	M	M	L	L	H	VL	L	L	M	VL	L
A3	VL	H	H	M	M	M	VH	L	M	VL	M	VL	L
A4	VH	M	VH	H	VH	H	VH	VL	L	VH	H	M	VH
A5	M	L	L	M	VH	L	H	H	H	H	L	L	L
A6	VH	L	VL	L	M	H	M	H	H	H	M	M	L
A7	VH	M	VH	VH	H	H	VH	M	M	H	M	H	M
A8	M	M	H	VH	VH	H	VH	VL	VL	H	H	M	VH
A9	M	VL	L	M	M	M	M	H	M	M	VH	L	VL
A10	VL	VL	VL	M	M	L	M	H	M	L	H	L	L
A11	H	H	H	M	H	M	VH	VL	VL	M	L	VH	H
A12	M	VH	VH	H	VH	H	H	M	M	M	M	M	H
A13	L	H	H	M	H	H	L	M	H	L	H	L	VL
A14	H	M	M	H	H	H	VH	M	M	M	M	M	VH
A15	H	H	M	H	H	H	H	L	M	H	VL	M	VH
A16	M	L	L	H	L	H	H	M	M	H	L	M	H
A17	VL	VL	M	M	VL	M	M	M	H	VL	M	L	VL
A18	H	M	VH	H	H	VH	VH	VL	VL	VH	H	M	H
A19	H	VH	H	VL	VL	L	H	VH	VH	M	VL	VL	L
A20	VL	H	M	VL	M	M	H	VH	VH	VL	L	VL	VL

**Table A3:** Rating of the alternatives by expert 2.

Model	UA1	UA2	UA3	UA4	UA5	UA6	UA7	UA8	UA9	UA10	UA11	UA12	UA13
A1	VL	VH	H	M	L	VL	H	VL	L	VL	M	VL	VL
A2	M	H	M	M	L	L	H	VL	L	L	M	VL	L
A3	VL	H	M	M	M	M	VH	L	M	VL	M	VL	H
A4	VH	M	H	H	H	H	VH	L	L	VH	H	M	H
A5	M	L	L	M	M	L	H	M	H	H	L	L	L
A6	VH	L	VL	L	H	H	M	VH	H	H	M	M	L
A7	VH	M	VH	VH	H	H	VH	M	M	H	M	H	M
A8	L	M	H	VH	VH	M	VH	VL	VL	H	H	M	VH
A9	H	VL	L	M	M	H	M	H	M	M	VH	L	VL
A10	VL	VL	L	M	M	L	M	H	M	M	H	L	L
A11	H	H	VH	M	H	M	VH	VL	VL	L	L	VH	H
A12	M	VH	H	H	VH	H	H	M	M	M	M	M	H
A13	L	H	H	M	H	H	L	M	H	L	H	M	VL
A14	H	M	M	H	H	H	VH	M	M	M	M	L	VH
A15	H	H	M	H	H	H	H	L	M	H	VL	H	VH
A16	H	L	L	H	L	H	H	M	M	H	L	M	H
A17	VL	VL	M	M	VL	M	M	M	H	VL	M	L	VL
A18	H	M	VH	H	H	VH	VH	VL	VL	VH	H	M	VH
A19	H	VH	H	VL	VL	L	H	VH	VH	M	VL	VL	M
A20	VL	H	M	VL	M	M	H	VH	VH	VL	L	VL	VL

**Table A4:** Rating of the alternatives by expert 3.

S/L	UA1	UA2	UA3	UA4	UA5	UA6	UA7	UA8	UA9	UA10	UA11	UA12	UA13
A1	L	VH	H	M	L	VL	H	VL	L	VL	M	VL	VL
A2	L	H	M	M	L	M	H	VL	M	L	L	VL	L
A3	VL	VH	H	M	M	L	VH	L	L	VL	M	VL	L
A4	VH	H	VH	H	VH	H	VH	VL	L	H	H	M	VH
A5	M	L	L	M	VH	L	H	H	H	H	L	L	L
A6	VH	L	L	L	M	H	M	H	H	H	H	M	L
A7	VH	M	H	VH	H	H	VH	M	M	H	M	H	L
A8	M	M	H	VH	VH	H	VH	VL	L	H	H	M	H
A9	M	VL	L	M	M	M	L	H	L	M	VH	L	VL
A10	VL	VL	VL	M	M	L	H	H	H	L	H	L	L
A11	H	H	H	M	H	M	VH	VL	VL	M	L	VH	H
A12	L	VH	VH	H	VH	H	H	L	M	M	M	M	H
A13	M	H	H	M	H	H	L	H	H	L	H	L	VL
A14	H	M	M	H	H	H	VH	M	M	M	M	M	VH
A15	H	H	M	H	H	H	H	L	M	H	VL	M	VH
A16	M	L	L	H	L	VH	H	M	M	VH	L	M	H
A17	VL	L	M	M	VL	L	M	M	H	VL	M	L	VL
A18	H	L	VH	H	H	VH	VH	VL	VL	VH	VH	M	H
A19	H	VH	H	VL	VL	L	H	VH	VH	M	L	VL	L
A20	VL	H	M	VL	M	M	H	VH	VH	VL	L	VL	VL
